# The Ser7 phosphorylation of RNA polymerase II-CTD is required for the recruitment of E3 ubiquitin ligase Asr1 and subtelomeric gene silencing

**DOI:** 10.1016/j.jbc.2025.110365

**Published:** 2025-06-11

**Authors:** Nikita Sudarshan, Mohd. Asalam, Ashutosh Kumar, Neha Singh, Adity Gupta, Ishita De, Sanjeev Kumar Singh, Kam YJ. Zhang, Md. Sohail Akhtar

**Affiliations:** 1 Biochemistry and Structural Biology Division, CSIR-Central Drug Research Institute, Lucknow, India; 2 Academy of Scientific and Innovative Research (AcSIR), Ghaziabad, India; 3 Laboratory for Structural Bioinformatics, Center for Biosystems Dynamics Research, Yokohama, Kanagawa, Japan; 4 Department of Data Sciences, Centre of Bio Medical Research, Lucknow, India

**Keywords:** E3 ubiquitin ligase, Asr1, RNA polymerase II, CTD, modeling

## Abstract

The carboxy terminal domain (CTD) of the largest subunit of RNA polymerase II (RNAPII) is composed of a tandem heptad sequence of Tyr1Ser2Pro3Thr4Ser5Pro6Ser7, which helps facilitate the transcription of all mRNA and the majority of noncoding RNA. The serines of RNAPII-CTD undergo differential phosphorylation, with Ser5 phosphorylation being predominant at the 5′ end, Ser2 phosphorylation toward the 3′ end, and Ser7 phosphorylation (Ser7P) present throughout the ORF during transcription. The phosphorylation of Ser2 and Ser5 coordinates the recruitment of proteins involved in the progression of transcription. The Ser7P has been shown to play a role in the processing and termination of small nuclear RNA transcription in both budding yeast and humans. Nevertheless, the effect of this phosphorylation mark on protein-coding genes remains unclear. This is despite the fact that substitution of Ser7 with phosphomimetic Glu does not support growth, and highly transcribed mRNA genes show high levels of this phosphorylation mark. In this study, we demonstrate that the interaction between E3 ubiquitin ligase Asr1 and RNAPII is influenced by the Ser7P in both *in vitro* and *in vivo* conditions. Asr1 appears to interact with the CTD in a distinct manner, where Ser7 is phosphorylated in the first heptad and Ser5 in the third heptad, involving key residues, such as Lys43, Arg48, Arg168, and Arg252. The Ser7P is important for the recruitment of Asr1 to RNAPII, and Ser7 mutation leads to the upregulation of subtelomeric genes. Ubc2 has been identified as the canonical ubiquitin-conjugating enzyme associated with Asr1.

The eukaryotic transcription necessitates that RNA polymerase II (RNAP II) gains access to DNA within the framework of chromatin (1, 2). The carboxy terminal tandem heptad repeat (YSPTSPS)n of the largest subunit of RNAPII (Rpb1) is referred as carboxy terminal domain (CTD), whose copy number generally increases with the complexity of the organism. CTD is dynamically modified where the phosphorylation and dephosphorylation allow for a vast combinatorial complexity of CTD primary structures, which orchestrate recruitment and dissociation of proteins involved in the transcription cycle (3, 4, 5). Although the presence of CTD phospho marks is known along the CTD length, the specific enrichment and pattern that helps the binding and dissociation of transcription regulatory proteins remains unclear (6). During transcription initiation and promoter escape, the Ser5 phosphorylation (Ser5P) helps in recruiting enzyme required for the capping of nascent transcripts (7, 8). During early elongation, Ser2 kinases act on the prephosphorylated Ser5, thus placing both Ser5P and Ser2 phosphorylation (Ser2P) marks, which recruit complexes required for transcription elongation and cotranscriptional processing (9, 10, 11). During the later phase, the CTD is primarily phosphorylated at Ser2 and helpful in recruiting factors essential for transcription termination, 3′ end formation, mRNA export and factors essential for recycling the RNAPII (12, 13). The Ser7 is phosphorylated at the 5′ end, and this mark remains high in both protein-coding and noncoding genes during the entire transcription cycle (14, 15). The role of Ser7 phosphorylation (Ser7P) is attributed to priming the function of other kinases, which in turn is helpful in the recruitment of RPAP2 phosphatase and the integrator complex for some small nuclear RNA (snRNAs; U1 and U2 snRNA) transcription in humans (16, 17, 18). The inhibition of Ser7 specific kinases markedly reduces the Ser7P mark on spliceosomal transcripts in *Saccharomyces cerevisiae* (19, 20, 21, 22, 23). The substitution of Ser7 with phosphomimetic Glu does not support growth, and hence, the essentiality of the hydroxyl group of Ser7 in cellular processes cannot be ruled out. Furthermore, a distinct pattern of all three Ser phosphorylations is observed in the protein-coding genes, and the role of Ser7P in mRNA transcription is consistent with the observation that highly transcribed genes show high levels of this phosphorylation mark; however, its role in mRNA transcription remains unclear.

The ubiquitin (Ub) and the Ub–proteasome system feature extensively in the regulation of gene expression, signaling histone modifications, mRNA export, and destruction of RNAPII. The protein ubiquitination is catalyzed by a three-enzyme cascade consisting of the Ub-activating enzyme (E1), Ub-conjugating enzyme (E2), and Ub ligase (E3) (24, 25, 26). Asr1 is an E3 Ub ligase and interacts with RNAPII-CTD in an Ser5P-dependent manner. Asr1-mediated nonproteolytic ubiquitylation of RNAPII leads to its inactivation. Asr1 ubiquitinates RNAPII at the transcribing genes near the telomere, leading to transcription termination and silencing of a subset of subtelomeric genes. Ubp3 deubiquitylase is known to deubiquitynate Rpb1 and function as an antisilencing factor in *S. cerevisiae*. Asr1 appears to interact with Ubp3, and the recruitment of this complex to CTD leads to the reversal of Rpb1 ubiquitination and the reassembly of the active RNAPII complex. Asr1 and Ubp3 play antagonistic roles in setting transcription levels from silenced genes (27, 28). Asr1 constitutively shuttles between the nucleus and cytoplasm, accumulates in the nucleus during stress, and is implicated also in the alcohol stress response (29).

We find that Asr1 has dual specificity for its interaction with RNAPII-CTD. The absence of Ser7 phosphorylation not only affects the *in vitro* and *in vivo* recruitment of Asr1 to RNAPII but also affects ubiquitination and subtelomeric gene silencing activities. We also identify the novel pattern of CTD that facilitates binding of Asr1 and the involved critical residues therein. Finally, we show that Ubc2 is an E2 of Asr1 for its ubiquitination properties. In a nutshell, Ser7 is essential for the recruitment of Asr1 to RNAPII-CTD, potentially playing a role in the silencing of a subset of subtelomeric genes in budding yeast.

## Results

### Ser7P is important for the recruitment of Asr1 and in subtelomeric gene silencing

The identification and characterization of transcription regulatory proteins provides valuable insight into the complexes that carry out essential cellular functions. The CTD-interacting proteins influencing the various stages of transcription have traditionally been identified by *in vitro* biochemical techniques and through *in vivo* genetic approaches. Later, the subunits of RNAPII were genetically tagged, and the associated proteins were identified through the analysis of pull-down samples or enriching the fractions specific to phosphorylation (30, 31, 32, 33, 34, 35). Nevertheless, the proteins whose interaction with RNAPII is influenced by Ser7, Tyr1, and Thr4 phosphorylations remain inadequately investigated. It is well known that the Ser7P mark remains high throughout the body of protein-coding gene, whereas the Ser5P peaks only at the 5′ end and the signal steadily decreases toward the poly-A site (4). The occupancy of Asr1 is noted throughout the entire length of the gene (27), indicating a significant overlap in the occupancy profile of Asr1 with Ser7P. Hence, the role of Ser7 in the recruitment of Asr1 cannot be ruled out.

**Figure 1:**
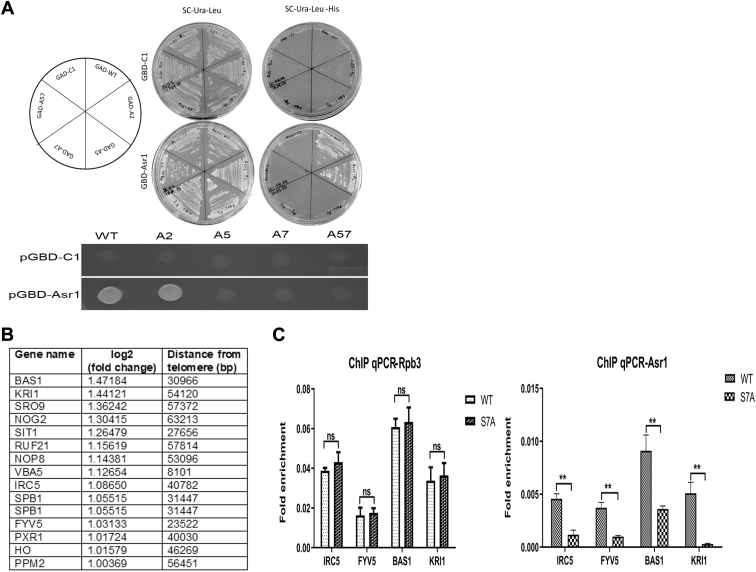
**Asr1 interacts with Ser7P of RNAPII-CTD for subtelomeric gene silencing**. *A,* the *left panel* shows the streaking pattern according to which the pJ69-4A yeast strain was cotransformed with respective GAD-CTDs and GBD-Asr1. The *central panel* streaks indicate the host strain being selected only for the presence of the expressions of both the plasmids. The *right panel* streak indicates the selection for the reporter gene expression because of the interaction between Asr1 and CTDs. The *lower panel* shows spotting from the above plate (*bottom right*) where cells grew optimally in the WT and Ser2 mutant but not in the case of Ser5 or Ser7 or both the mutants. *B,* the subtelomeric genes (median distance from the telomere of less than 60 kb) differentially expressed more than twofold between WT and Ser7 mutant cells. *C,* the Ser7 mutation of CTD affects subtelomeric gene silencing by Asr1. ChIP analysis of Asr1 across the subtelomeric genes where the distribution of total Rpb3 (*left panel*) and endogenous FLAG-tagged Asr1 (*right pane*l) was examined in the WT and Ser7 mutant RNAPII cell. All the signals were normalized to the input DNA. Shown is the mean of three ChIP experiments ± SEM. ChIP, chromatin immunoprecipitation; CTD, C-terminal domain; RNAPII, RNA polymerase II; Ser7P, Ser7 phosphorylation.

To investigate the necessity of Ser7P for the interaction between Asr1 and CTD, we carried out a modified yeast two-hybrid analysis, where a phosphorylation-dependent binding of proteins to the CTD is observed for the reporter gene expression (36). The binding of Asr1 to the native CTD (Ser2Ser5Ser7 or WT) or CTD with a single serine mutation (Ser2Ala or A2; Ser5Ala or A5; and Ser7Ala or A7) or double mutation (Ser5Ala + Ser7Ala or A57) was analyzed (Fig. 1*A*). The cells expressing GBD-Asr1 (Asr1 cloned downstream to Gal4-binding domain) grew on medium lacking Ura, Leu, and His, when coexpressed with GAD-CTDs (WT and A2), depicting the presence of the interaction between GAD-CTDs and GBD-Asr1. The WT and A2 cells of the Petri dish segment were spotted from the serial dilutions (Fig. S1*A*). The observed similar growth in both the cases indicates that Ser2P does not play a direct role in the interaction with CTD. The absence of comparable growth in the cases of A5, A7, or A57 double mutants suggests a disruption in the interactions with GBD-Asr1, highlighting the significant role of Ser7P in facilitating the binding of Asr1 to RNAPII-CTD.

Asr1 is recognized for its ability to ubiquitinate the lysines of both full-length and truncated Rpb1 in a manner dependent on Ser5P. The *in vitro* activity of Asr1 also shows the formation of unanchored poly-Ub chain and a minor nonspecific self ubiquitination activity (27). Since the Ser7P appears crucial in the recruitment of Asr1, we investigated whether the Ser7 mutation has any role in its recruitment led ubiquitination properties. The *in vitro* ubiquitination of glutathione-*S*-transferase (GST)-CTD, which includes four repeats of either the YSPTSPS or YSPTSPA heptads phosphorylated by kin28 (which phosphorylates Ser5 and Ser7), demonstrates a notable difference in ubiquitination, attributed to the Ser7 mutation (Fig. S1*B*).

A comparative transcriptome analysis of budding yeast expressing either the WT Asr1 or a catalytically inactive RING finger mutant Asr1 has demonstrated that numerous induced genes are situated within approximately 50 kb of the telomere. Asr1 interacts with telomere-proximal chromatin, and the disruption of its Ub-ligase activity, or modifications to the ubiquitination sites of Rpb1, induces the transcription of silenced gene sequences (28). To see if the Ser7 plays any role in the recruitment of Asr1 and the expression of subtelomeric genes, RNA-Seq analysis was carried out of budding yeast strains, expressing either the native or the mutant form of Ser7 in RNAPII-CTD. A significant number of induced genes was observed in the subtelomeric region as a consequence of the Ser7 mutation (Fig. 1*B* and Table S1). This may likely be due to the impairment of Asr1 recruitment to the RNAPII, and hence, we carried out chromatin immunoprecipitation–quantitative PCR (ChIP–qPCR) of a few induced genes located within 60 kb of telomere (Fig. 1*C*). The mutation in Ser7 appears to influence the function of Asr1 in subtelomeric gene silencing, as evidenced by a significant reduction in Asr1 occupancy across the chosen genes, whereas the occupancy of Rpb3 remains unchanged. The observed decrease of Asr1 occupancy in the S7A cells implies that the residue is important for the recruitment of Asr1 to RNAPII at the telomere-proximal genes for the subtelomeric gene silencing.

### Asr1 structure and its interaction with CTD

Many proteins are known to interact with the RNAPII-CTD; however, the stereochemical basis underlying recognition remains poorly understood (4, 37). The structural analysis of CTD-interacting proteins or domains with the phosphorylated CTD is quite restricted and has only been successfully achieved for a limited number of proteins, including Pcf11, Rtt103, Pin1, and Nrd1. In crystal structures, the flexible CTD heptad is not visible because of its inherent mobility; thus, a bioinformatics approach is required to comprehend the CTD–protein interaction and function (38, 39, 40).

**Figure 2:**
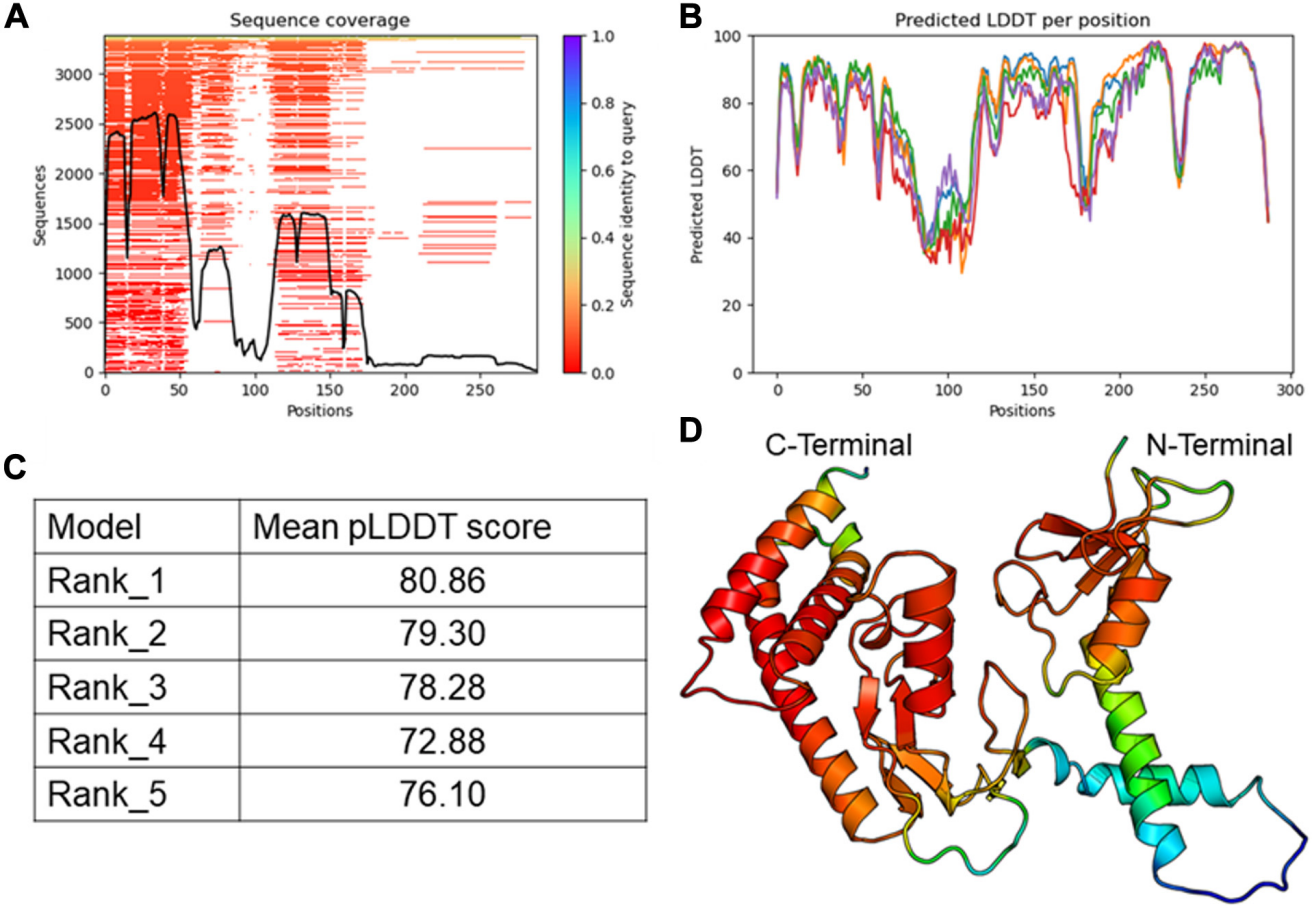
**Structure of Asr1**. *A,* sequence coverage in the multiple sequence alignment. *B,* pLDDT score across the full protein sequence. *C,* mean pLDDT scores for five predicted models. *D, cartoon* depiction of the predicted Asr1 structure colored according to the pLDDT score (*red* for *high* and *blue* for *low*). pLDDT, predicted local-distance difference test.

The structure of Asr1 has not been elucidated, and also no suitable structural homology of the enzyme is available. The 3D structure of Asr1 was predicted utilizing the AlphaFold software (41). The structure prediction showed good sequence coverage across multiple sequence alignments, indicating the effectiveness and accuracy of the model in capturing sequence-dependent structural features (Fig. 2*A*). The predicted structures of Asr1 exhibited reasonable model confidence, with a mean predicted local-distance difference test (pLDDT) score exceeding 75 for all models, reflecting a satisfactory degree of accuracy (Fig. 2, *B* and *C*). The pLDDT scores reflected a high level of confidence in the structural predictions at both the N- and C-terminal regions of the protein, with pLDDT scores remaining robust despite differing levels of sequence coverage (Fig. 2*D*). Notably, the C-terminal region displayed high pLDDT scores even with relatively lower sequence coverage than the N-terminal region. This suggests that the available evolutionary information, although limited, was significant to enable reliable predictions in this region. The model showed lower prediction accuracy for the segment spanning amino acid residues 70 to 120, where the pLDDT scores were notably reduced. Overall, the AlphaFold predicted structure of Asr1 reveals a modular architecture comprising three distinct domains. At the N terminus, there is a compact RING domain characterized by a zinc-finger fold, which is commonly associated with E3 Ub-ligase activity. Following this is the PHD domain, which likely features a characteristic zinc-binding fold that may play a role in chromatin interaction. At the C-terminal region, the model predicts a structured CTD-binding domain (CBD), with an interface capable of interacting with the heptad repeats of the RNAPII-CTD (27). The overall structural organization of the predicted Asr1 model supports its function in ubiquitination and transcriptional regulation.

**Figure 3:**
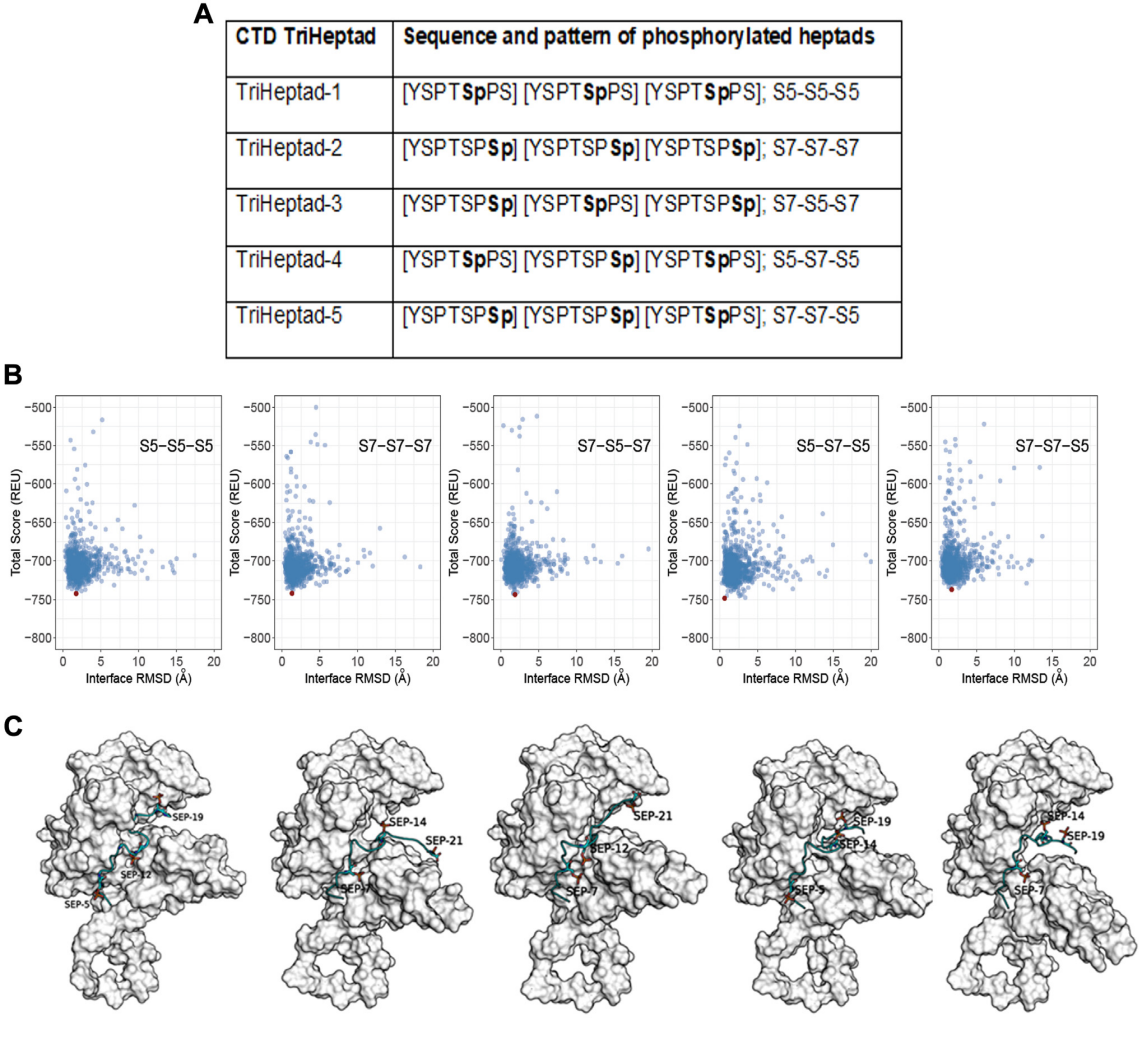
**Docking and simulations of Asr1 with CTD TriHeptads**. *A,* sequences of TriHeptad used for computational analysis. Sequences were numbered from 1 to 21 for the simulation. *B,* scatterplots showing the correlation between “total_score” from Rosetta and RMSD at the interface between the Asr1 and TriHeptad. These plots are derived from protein–protein docking simulations of Asr1 with CTD TriHeptads. The model displaying the *highest* interaction energy is marked with a *red dot*. *C,* visual representations of the predicted complex structures formed by docking Asr1 with TriHeptad. Asr1 is illustrated with a *white surface*, whereas TriHeptads are depicted using a *cyan ribbon*. Phosphorylated serines are highlighted in *stick form*. CTD, C-terminal domain.

The top-ranked predicted structure of Asr1 derived from AlphaFold was utilized to model its interaction with the TriHeptads, which are more likely to interact with Asr1 (Fig. 3*A*). To gain a deeper understanding of the interaction specificity, a multistep protein–protein docking approach was used to generate the complex structures of Asr1 with TriHeptads (Fig. S2) (42, 43). The scatter plot analysis of the Rosetta “total score” and “interface RMSD” illustrated the presence of well-defined docking funnels across all TriHeptads, indicating successful convergence toward energetically favorable complex configurations (Fig. 3*B*, shown as *red points*). The structures exhibiting the lowest Rosetta “total score” indicated that all TriHeptads bind to Asr1 in a similar manner, occupying the region between the N-terminal and C-terminal domains (Fig. 3*C*). This consistency suggests a specific interaction pattern that stabilizes the complex.

Subsequently, we expanded our investigation by performing molecular dynamics (MD) simulations to gain a deeper understanding of the interactions between Asr1 and CTD TriHeptads (44, 45). Using the mdciao and GetContacts Python libraries, we conducted a contact analysis to explore the interactions between the phosphorylated CTD and Asr1. This analysis revealed a consistent pattern of significant contacts crucial for mediating the interaction. Notably, the phosphorylated serine (SEP) residues of the TriHeptads were identified as central to these interactions. Previously, we reported that Arg, Asn, and Lys are the primary residues interacting with SEPs, based on the analysis of crystal structures containing SEPs (46). These phosphorylated serines tend to interact with positively charged amino acids, likely because of electrostatic attraction exerted by the negatively charged phosphate groups. Our MD simulation of the Asr1 and TriHeptad complex structures also indicated that Arg and Lys are the primary interacting residues with high occupancy, especially in the case of TriHeptad-1 (S5–S5–S5) and TriHeptad-5 (S7–S7–S5) (Figs. S3 and S4).

**Figure 4:**
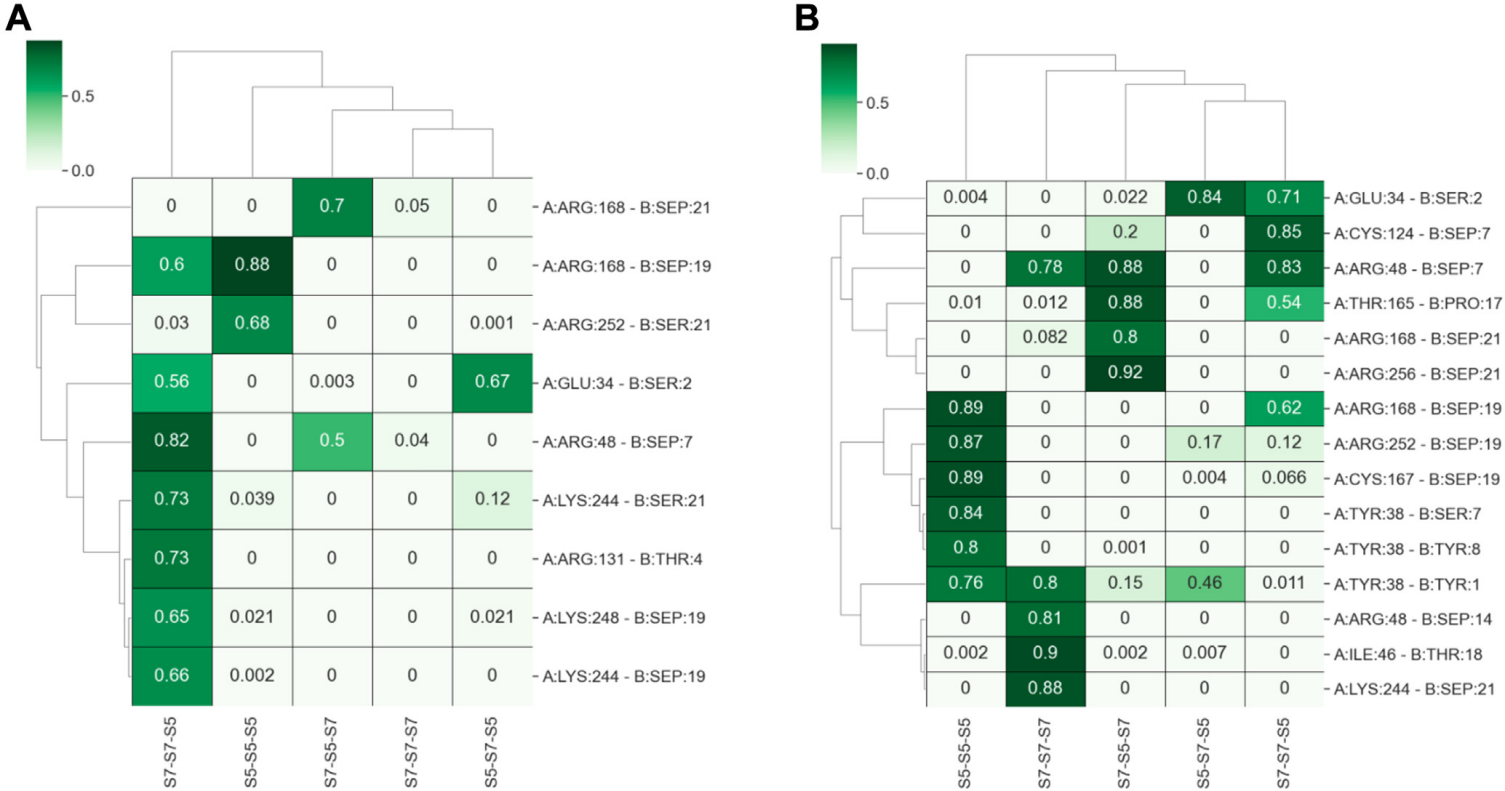
**Probable contacts between Asr1 and TriHeptads**. Heatmaps depicting the frequency of hydrogen bonds (*A*) and van der Waals contacts (*B*) between TriHeptads and Asr1 throughout a 500 ns MD trajectory. Displayed interactions meet or exceed an occupancy threshold of 0.6 for hydrogen bonds and 0.8 for van der Waals contacts on a scale of 0 to 1. MD, molecular dynamics.

**Figure 5:**
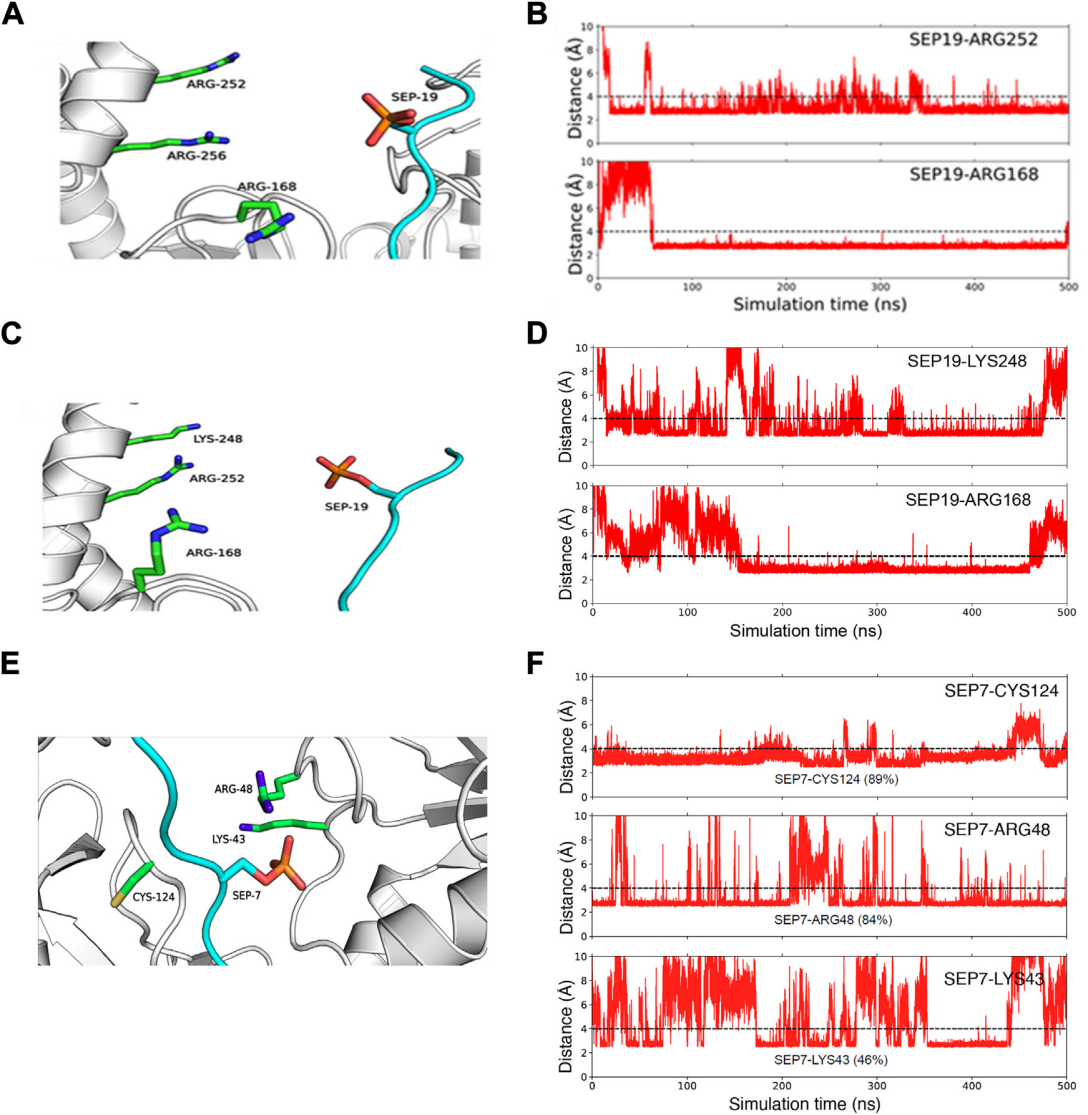
**Interaction between Asr1 and TriHeptads**. *A,* close-up view of the docking-predicted complex structure of Asr1 and S5–S5–S5. *B,* line plot depicting the distance between SEP19 of S5–S5–S5 and two interacting residues throughout a 500 ns MD trajectory. *C,* close-up view of the docking-predicted complex structure of Asr1 and S7–S7–S5. *D,* line plot depicting the distance between SEP19 of S7–S7–S5 and the two interacting residues, Arg168 and Arg248. *E,* close-up view of the docking-predicted complex structure of Asr1 and S7–S7–S5. *F,* line plot depicting the distance between SEP7 of S7–S7–S5 and the three interacting residues, Cys124, Arg48, and Lys43. MD, molecular dynamics; SEP, phosphorylated serine.

The S5–S5–S5 and S7–S7–S5 exhibited higher contacts with Asr1 residues compared with other TriHeptads. Specifically, S5–S5–S5 primarily interacted with Lys37, Tyr38, Cys167, Arg168, Arg252, and Arg256. Similarly, S7–S7–S5 interacted with Lys43, Arg48, Cys124, Arg168, Lys244, and Lys248 (Fig. 4). Among these interactions, the hydrogen bond between SEP19 (serine phosphorylated at the 19th position in the third heptad) of S5–S5–S5 and Arg168 was found to be the most prominent contact, occurring in over 80% of the MD trajectory frames (Figs. 5*A* and 4*A*). Interestingly, in the docking-predicted complex structure of Asr1 with S5–S5–S5 and the initial 50 ns of MD simulation, SEP19 was located slightly farther away. However, after the initial frames, the formation of this hydrogen bond was observed, which remained stable for the rest of the simulation, as noticed by measuring the distance between SEP19 and Arg168 throughout the 500 ns MD simulation (Fig. 5*B*). This highlights the importance of MD simulations in studying the dynamic nature of protein–peptide interactions. In addition, another Asr1 residue, Arg252, played a crucial role in stabilizing the interaction between Asr1 and S5–S5–S5.

A consistent hydrophobic contact was observed between SEP19 and Arg252, occurring with a frequency of more than 0.8 (Figs. 4*B* and 5*A*). This contact remained quite stable throughout the 500 ns MD simulation. Specifically, the distance between Arg252 and SEP19 was below 4 Å in approximately 88% of the MD frames, underscoring the persistent and significant nature of this interaction (Fig. 5*B*). Similar to the pattern observed in the case of S5–S5–S5, Arg168 was also a crucial residue for interaction with S7–S7–S5. Here, SEP19 interacted with Arg168 by forming both hydrogen bonds and hydrophobic contacts (Fig. 4). Apart from Arg168, SEP19 also interacted with Lys248, mainly *via* hydrogen bonding. Both these contacts were consistently observed in the majority of the frames of the 500 ns MD trajectory (Fig. 5, *C* and *D*). In addition to the SEP19, the SEP at position 7 of the first heptad (SEP7) also stabilized the interaction between Asr1 and S7–S7–S5. Here, SEP7 interacted with Lys43, Arg48, and Cys124 by forming both hydrogen bonds and hydrophobic contacts (Figs. 5, *E* and *F* and Fig. 4). The contact with Arg48 and Cys124 remained stable in most of the frames of the MD trajectory. The simulation studies suggest the Asr1 interacts with the CTD in a unique pattern, where both the Ser7 and Ser5 of the alternate heptad play important roles.

### Key residues of Asr1 interacting with CTD

**Figure 6:**
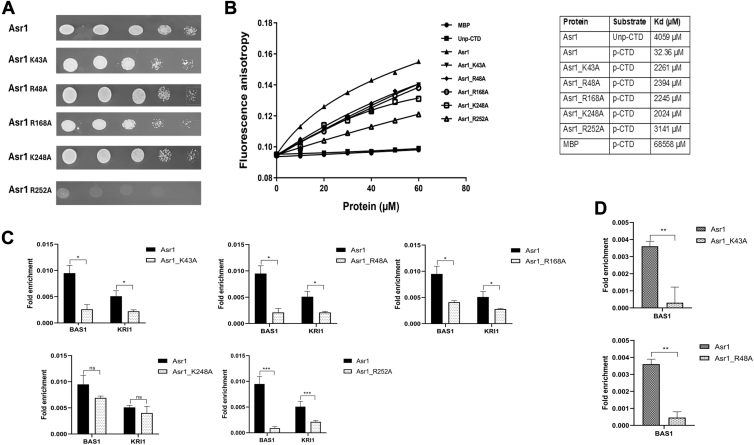
**Binding characteristics of Asr1 to the CTD**. *A,* Y2H analysis where the pJ69-4A strain was cotransformed with GAD-CTD and GBD-Asr1 or with its various mutants. The growth because of the transcription of a His3 reporter, resulting from the interaction between Y2H fusion proteins, was analyzed. *B,* the fluorescence anisotropy measurements, where 2 μM of FAM-labeled unphosphorylated CTD (Unp-CTD or YSPTSPS–YSPTSPS–YSPTSPS) and phosphorylated CTD (p-CTD or S7–S7–S5) were titrated against increasing concentrations of Asr1, its mutants and MBP to determine the binding efficiency. *C,* the occupancy of Asr1 mutants across the induced subtelomeric genes in WT cells. *D,* the occupancy of Asr1 mutants in Ser7 mutant cells. All the signals were normalized to the input DNA. Shown is the mean of three ChIP experiments ± SEM. ChIP, chromatin immunoprecipitation; CTD, C-terminal domain; MBP, maltose-binding protein; Y2H, yeast two-hybrid system.

To see the *in vivo* efficiency of Asr1 and its mutants in binding to CTD, we carried out yeast two-hybrid system as detailed previously (Fig. 6*A*). The critical residues of Asr1 that interact with CTD were substituted with alanine and subsequently cloned downstream to GBD, resulting in constructs GBD-Asr1_K43A, GBD-Asr1_R48A, GBD-Asr1_R168A, GBD-Asr1_K248A, and GBD-Asr1_R252A. The pJ69-4A vector was cotransformed with the native GAD-CTD and various GBD-Asr1 mutants. The cells expressing the native GBD-Asr1 exhibited optimal growth in a medium devoid of uracil, leucine, and histidine. The mutants GBD-Asr1_K43A, GBD-Asr1_R48A, GBD-Asr1_R168A, and GBD-Asr1_K248A did not show significant change of growth in the spotting assay. However, in a similar condition, GBD-Asr1_R252A did not grow optimally, suggesting a total absence of interaction between Asr1 and CTD. The growth of cells was further analyzed in the liquid media to better assess the change in growth (Fig. S5*A*). A notable difference in growth was observed also in the case of Asr1 mutants as compared with the WT strain.

The binding properties of the CTD-interacting proteins are extensively looked upon by fluorescence anisotropy assay (39, 46). We evaluated the binding efficiency of Asr1 with all five TriHeptads mentioned in Figure 3*E*, having the patterns of S5–S5–S5, S7–S7–S7, S5–S7–S5, S7–S5–S7, and S7–S7–S5 (Fig. S5*B*). The titration of heptads with increasing concentrations of Asr1 indicates a preferential binding of Asr1 to the S7–S7–S5 heptad in comparison to the other peptides. The other peptides also show significant associations with Asr1, likely because of the abundant molecular interactions between phosphorylated TriHeptads and Asr1 in the vicinity of the binding pocket. To see the impact of key residues of Asr1 on its interaction with the S7–S7–S5, the binding efficiency of various recombinantly expressed Asr1 mutants (Asr1_K43A, Asr1_R48A, Asr1_R168A, Asr1_K248A, and Asr1_R252A) was analyzed (Fig. 6*B*). All mutants exhibited an increased dissociation constant (*K*_*d*_) compared with the native Asr1, indicating their involvement in the interaction with the CTD. The control maltose-binding protein (MBP) showed insignificant binding to the CTD. These findings imply that a single residue may not be a sole determinant of Asr1–CTD interaction and is being facilitated through multiple interactions.

To determine the impact of Asr1 mutations on subtelomeric gene silencing, we examined their occupancy on genes whose expression is induced because of Ser7 mutation. The ChIP–qPCR analysis of FLAG-tagged Asr1 and its mutants revealed a significant loss of occupancy for all mutants, with the exception of Asr1_K248A on the BAS1 and KRI1 genes (Fig. 6*C*). These findings indicate that Asr1 occupies subtelomeric genes through critical interactions involving Lys43, Arg48, Arg168, and Arg252A in the WT cells. In addition, the more notable reduction in occupancy of Asr1_K43A and Asr1_R48A was observed in the case of Ser7 mutant cells, which strongly implies that the residues play a significant role in the disruption of subtelomeric gene silencing (Fig. 6*D*). The aforementioned studies suggest that the SEP7 makes key interactions with Lys43 and Arg48, whereas SEP19 interacts with Arg252 and Arg168.

### Ubc2 is an E2 for Asr1

**Figure 7:**
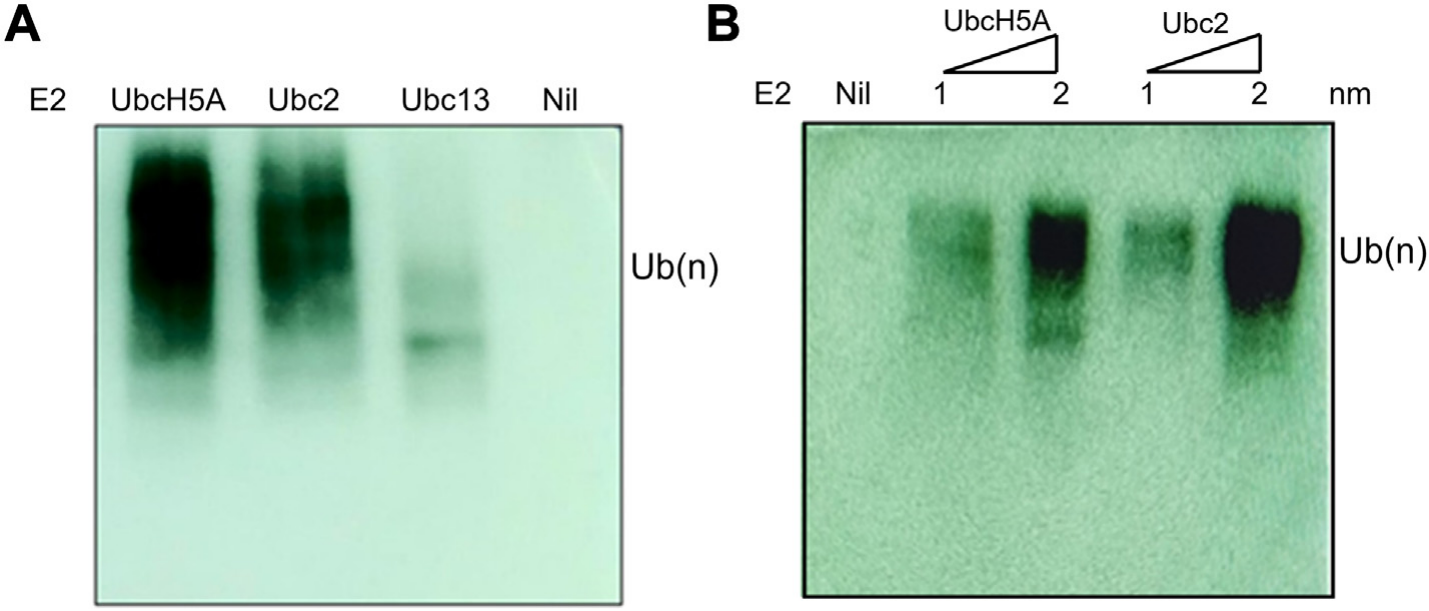
**Ubc2 is an E2 of Asr1**. *A,* the *in vitro* polyubiquitination assay of recombinant MBP-Asr1 using commercial E2 (recombinant UbcH5ba), yeast Ubc2 and Ubc13 (4 μM each), and detection using α-Ub antibody. *B,* the *in vitro* polyubiquitination assay of recombinant MBP-Asr1 at increasing concentrations of UbcH5a and Ubc2. MBP, maltose-binding protein; Ub, ubiquitin.

Ub-conjugating enzymes (E2s) occupy the center of the E1–E2–E3 cascade and are essential to the enzymatic process that facilitates the attachment of Ub to a substrate. In budding yeast, a single E1 enzyme activates Ub, whereas 13 E2 enzymes (Ubc1–Ubc13) participate in the conjugation of Ub or Ub-like proteins. Ub ligases (E3s) represent the largest group, comprising over 100 proteins that are integral to the ubiquitination process, providing both selectivity and specificity. Of 13 E2s, Ubc9 and Ubc12 do not conjugate Ub, and among 11 canonical E2s, Ubc3 is an essential enzyme (21, 23). To determine the canonical E2 for Asr1, we conducted a literature review, which led us to focus on two potential E2 enzymes, Ubc2 and Ubc13, known for their roles in DNA repair and transcription in budding yeast. The TAP-tagged Ubc2 and Ubc13 were purified using standard protocols (Fig. S6*A*). The autoubiquitination assay of Asr1 was carried out as detailed before using UbcH5A (a member of human E2 Ub -conjugating enzymes and known to act as an E2 for Asr1 in the *in vitro* ubiquitination assay) along with the purified Ubc2 and Ubc13. The results indicated that Asr1 underwent autoubiquitination in the presence of Ubc2 but not with Ubc13 (Fig. 7*A*). In addition, increasing concentrations of Ubc2 led to a corresponding rise in ubiquitination (Fig. 7*B*). We further asked whether the Ubc2 also influences the Asr1 activity for the CTD-led ubiquitination, and hence, an *in vitro* ubiquitination of GST-CTD was carried out (Fig. S6*B*). The observed ubiquitination of GST-CTD suggests that the Ubc2 may be involved in the CTD-led ubiquitination in the cell.

## Discussion

The CTD of RNAPII plays an important function during mRNA biogenesis where it couples transcription with RNA processing during the transcription cycle. The exact role of each CTD heptad modification is difficult to determine because of the essentiality and pleiotropy of the factors involved as well as the probable additional substrates of the kinases and phosphatases that play a major role in gene expression. Unlike in *S. cerevisiae* and humans, Ser2P is not essential in *Saccharomyces pombe* and in conjunction with Ser7P, its role is implicated in phosphate homeostasis and in promoting heterochromatin formation by linking noncoding RNA. The S7A substitution suppressed the mating defect of an Rpb1-S2A mutant in fission yeast (47, 48, 49, 50, 51, 52).

In addition to the reversible phosphorylation of Ser2 and Ser5, we have now begun to understand the roles of Tyr1, Thr4, and Ser7 in the transcriptional regulation of both budding yeast and humans. It remains enigmatic what direct role is played by the Ser7P in mRNA transcription in budding yeast. Notably, the Ser7P mark is consistently present throughout the coding regions of protein-coding genes, whereas Ser5P is predominantly found at the 5′ end. This suggests that Ser7P may assist in retaining Ser5P-interacting proteins toward the 3' end. Furthermore, the Ser7P of the RNAPII is known to prime the activity of CTD kinases (Cdk9 and Cdk12), which in turn helps the recruitment of RPAP2 and the integrator complex to snRNA genes in humans, as well as involved in mRNA capping in yeast. In this context, Cdk7 is responsible for phosphorylating Ser5 and Ser7 at the 5′ end, with Ser7P being crucial for the subsequent Ser5P and Ser2 by additional kinases. Since the occupancy of Asr1 is throughout the body of genes, the enhanced binding specificity of Asr1 to the CTD is likely a result of the priming function of Ser7P (16, 17, 18). The role of Ser7P appears to be in conjunction with Ser5P to modulate the function of Asr1. The prosilencing action of Asr1 is attributed to its interaction with Ser5P of RNAPII-CTD. Conversely, Asr1 also interacts with the deubiquitinase complex (Upb3/Bre1) and recruits it to the RNAPII-CTD to antagonize its activity by deubiquitylating the Rpb1 to restore transcription. A mutation of Ser5 to alanine disrupts the interaction of Asr1 with the CTD and its subsequent ubiquitination activity (27). We also observed the reduced ubiquitination of GST-CTD when Ser7 was substituted with Ala.

The phosphorylation of the CTD is consistently distributed along its entire length, typically featuring one phosphorylation mark per heptad. The adjacent heptads can be differently phosphorylated, leading to a wide range of coexisting phosphosites in monoheptad and diheptad repeats. Therefore, it is likely that any combinatorial phosphorylation site recognition by CTD-interacting proteins probably occurs over multiple repeat units (6, 53). The current understanding of the stereochemical basis of CTD recognition by CBD is mostly based on a few 3D structures of proteins, such as Pcf11, Rtt103, Nrd1, Cgt1, and Ess1 bound to the phosphorylated heptads (either Ser5 or Ser2 or both on the single heptad) or through *in vitro* binding assays (37, 38, 39, 40). The phosphorylation of Ser7 is anticipated to not only modify the chemical structure of the CTD but also enhance or limit the conformational flexibility of the motif, potentially influencing recognition. The genome-wide profiles of Ser5P and Ser7P indicate that these two marks are positioned on adjacent repeats; however, their pattern, which facilitates the interaction of CBDs, is not known (3, 4, 5, 6). A deeper understanding of the binding mechanisms of CBD concerning Ser7P or other residues is likely to provide additional insights into the mechanism of CTD function in mRNA biogenesis. How the interaction between Asr1 and the flexible CTD heptad of RNAPII is facilitated is investigated through the bioinformatics approach. The MD simulation of Asr1 interacting with the CTD TriHeptads phosphorylated at Ser5 (S5–S5–S5) or Ser7 (S7–S7–S7) or both at Ser5 and Ser7 (S5–S7–S5 and S7–S5–S7) or in a heptad where two distant heptads were phosphorylated differently (S7–S7–S5) gave a novel insight into the binding pattern. It is determined that Ser7P and Ser5P are critical for interacting with various residues of Asr1. Notably, Arg252 and Arg168 of Asr1 are significant in their interaction with Ser5P of the third heptad in either S5–S5–S5 or S7–S7–S5 configurations. Similarly, the Lys43 and Arg48 are observed to form a stable contact with Ser7P of the first heptad of S7–S7–S5. The mutation of the key residues followed by the binding assay and occupancy at subtelomeric genes highlighted the crucial role of Lys43, Arg48, Arg168, and Arg252 in promoting the interaction between Asr1 and the CTD. Hence, it is likely that the Asr1 interacts with CTD in a novel manner where Ser7 is phosphorylated in the first heptad and Ser5 is phosphorylated in the third heptad sequence. Due to the structural similarity among E2s and the working of E2s in pairs for a given E3 *in vivo*, the molecular determinants for the function of E2s are not very clear (26). However, we observed that one of the E2 (Ubc2) independently facilitate the *in vitro* ubiquitination activity of Asr1.

## Experimental procedures

### Plasmids and strains

Plasmids and yeast strains used in this study are listed in Table S2. Yeast E1, human E2 (UbcH5a), GST Ub, and Mg–ATP were purchased from Boston Biochem.

### Preparation of proteins

The Asr1 gene (867 bp) was amplified from the genomic DNA of *S. cerevisiae* (S288C). The amplified fragment and pMAL-c4X vector were digested with BamHI and HindIII, followed by the ligation and transformation into the DH5-α cell to obtain the positive clone. The mutants of Asr1 (K43A, R48A, R168A, K248A, and R252A) were generated using Q5 Site–Directed mutagenesis system (New England Biolabs) using suitable primers (Table S3), and the amplification conditions were used as specified in the kit. The homogeneity of the sequence was confirmed by DNA sequencing before optimizing their expression. The MBP, MBP-Asr1, or MBP-Asr1 mutants were expressed in BL21(DE3) cells and purified by amylose resin chromatography and were processed as per the user’s manual (New England Biolabs).

### Construction of 3X-FLAG-tagged Asr1 mutants in the WT and Ser7 mutant cells

In order to construct the strains, the Asr1 gene was first deleted from both WT-RNAPII and S7A-RNAPII cells. For deleting the Asr1 gene, the standard PCR-based homologous recombination method was used, and the gene was replaced with a uracil marker. The primer sequences are listed in Table S4. For C-terminal 3X-FLAG tagging of the Asr1 gene or its mutants, the following strategy was employed. The 5′HR of the Asr1 gene was overlapped to the ORF of Asr1 gene and its mutants as desired. The resulting cassette (5′HR + Asr1) or (5′HR + Asr1 mutants) was then overlapped to the 3X-FLAG tag by overlapping PCR. The overlapped fragment (5′HR-Asr1-3X FLAG) and pFA-hph-MX6 vector were double digested with BamH1/Asc1 and ligated. The 3′HR was PCR amplified and ligated into the pFA-hph-MX6 vector. The final construct was double digested with BamH1 and Spe1, and the resulting cassette (5′HR-Asr1-3XFLAG-HYG-3′HR) was transformed into WT or S7A Rpb1 (Asr1Δ) yeast strain, and positive colonies were selected on YPD + hygromycin plate. The positive colonies were screened by PCR, and the expression of the FLAG tag was confirmed by WWestern blotting. The primer sequences are listed in Table S5.

### Ubiquitylation assay

*In vitro* ubiquitination assay was carried out as described before (27). Briefly, in a 30 μl reaction volume with a final concentration of 0.1 μM yeast E1; 1 to 4 μM human or yeast E2s; 1 μM MBP or MBP-Asr1; 2 mM Mg–ATP; 50 mM Tris-Cl pH 7.5; 2.5 mM MgCl_2_; 2 mM Z_n_SO_4_; and 0.5 mM DTT. The reactions were incubated at 30°C for 90 min and were stopped by adding SDS-PAGE loading and analyzed through Western blotting using standard anti-UUb antibody.

### Yeast two-hybrid system

The plasmids pGAD-S2S5S7 (14 repeats), pGAD-A2S5S7 (14 repeats), pGAD-S2A5S7 (14 repeats), and pGAD-S2S5A7 (14 repeats) were constructed as described previously (54). The coding sequence for Asr1 and its mutants (K43A, R48A, R168A, K248A, and R252A) was subcloned from pMAL-c4X vector clones into pGBDU-C1 between BamH1 and Sal1, respectively. The assay was performed by cotransforming pJ69-4A strain with GAD-CTD plasmids and GBD-Asr1 or with GBD-Asr1 or its mutants. The growth was assayed either by streaking or by spotting a 10 μl aliquot of serial 10-fold dilutions on Synthetic Dropout (-Ura-Leu-His) plates and incubated at 30 °C for 48 h before being photographed.

### Fluorescence anisotropy

For binding experiments, MBP, MBP-Asr1, and MBP-Asr1 mutants were titrated into a reaction mixture containing buffer (25 mM Hepes [pH 8], 100 mM NaCl, 1 mM EDTA, and 1 mM DTT) at 25 °C, supplemented with 2 μM of unphosphorylated CTD (FAM-YSPTSPS YSPTSPS YSPTSPS), and phosphorylated CTDs (FAM- YSPTSpPS-YSPTSpPS-YSPTSpPS/S5–S5–S5, FAM- YSPTSPSp-YSPTSpPS-YSPTSPSp/S7–S7–S7, FAM- YSPTSpPS-YSPTSPSp-YSPTSpPS/S5–S7–S5, FAM- YSPTSPSp-YSPTSpPS-YSPTSPSp/S7–S5–S7, and FAM- YSPTSPSp-YSPTSPSp-YSPTSpPS/S7–S7–S5). Measurements were performed in a T-configured fluorescence spectrometer. Data were fitted to the cubic equation applying nonlinear regression one site total binding mode in GraphPad Prism 5 (GraphPad Software, Inc), as described previously (39, 46).

### ChIP and qPCR

ChIP was carried out as described previously (27, 54). For the detection of 3X FLAG Asr1 occupancy in WT and S7A of Rpb1, parallel ChIP assays were performed using the 8HCLC (Thermo) polyclonal antibody. The signals from ChIP DNA obtained were quantified by real-time PCR. The signals from ChIP DNA from the 3X FLAG Asr1 strain were normalized to the corresponding signals (per primer set) from the untagged yeast (BY4741). Signals are presented as a percentage of immunoprecipitation efficiency (ChIP/input DNA). Data from ChIP experiments are the average (SEM) of three distinct ChIP assays. The sequences of primers used for the ChIP–qPCR study are listed in Table S6.

### Prediction of Asr1 3D structure and modeling

The 3D structure of Asr1 was predicted using AlphaFold (41). We acquired the AlphaFold2 software from its official Github repository (https://github.com/deepmind/alphafold) and set it up on a Linux workstation equipped with an NVIDIA Quadro RTX8000 GPU. For the structure prediction, we utilized the "full_dbs" db_preset setting along with AlphaFold's "monomer" configuration. Five models were generated, and the model with the highest pLDDT score—a confidence metric from AlphaFold was selected. The pLDDT scores were extracted using a modified "visualize_alphafold_results.py" Python script available at https://github.com/jasperzuallaert/VIBFold.git.

To simulate the interaction between Asr1 and the hyperphosphorylated CTD, we created four sequences of CTD TriHeptads, each featuring a SEP at varying positions (TriHeptad, 1–5) (Fig. 3*A*). The 3D structure of the CTD TriHeptad was taken from the crystal structure of the mediator head module from *S. cerevisiae* bound to the CTD of RNAPII, Rpb1 subunit (Protein Data Bank code: 4GWQ) (55). Modifications were then made to serine residues in CTD TriHeptad to reflect their phosphorylated states, according to the designed sequences. To predict the interaction between Asr1 and the CTD, we applied a two-step docking procedure. Initially, we used the ClusPro protein–protein docking tool to predict the complex of the nonphosphorylated TriHeptad with Asr1 (42). Subsequently, the phosphorylated TriHeptads were superimposed on these initial ClusPro models, and these configurations were refined using the protein–protein docking protocol of Rosetta, version 3.9 (43, 56, 57). The docking was executed employing the RosettaScript "high-resolution docking" XML protocol (58), focusing on a local search with adjustments limited to 3 Å and 8°. The REF2015 scoring function (59, 60) was utilized to evaluate the docking outcomes. Approximately 1000 poses were generated, prioritized first by Rosetta’s “total score,” and then the 100 lowest energy models were further refined based on Rosetta’s “interface_delta” score, which is equivalent to interaction energy. The model displaying the lowest “interface_delta” for each TriHeptad was then chosen for more detailed examination through MD simulations.

### MD simulation

MD simulations were performed using the phosphorylated TriHeptad structures complexed with ASR1, as predicted by protein–protein docking. Each simulation system was set up by solvating with TIP3P water molecules within a dodecahedral box and subsequently neutralized by the addition of Na^+^ or Cl^-^ ions. Parameters for the protein, solvent, and ions were specified using the CHARMM27 all-atom force field (61), whereas SEP parameters were obtained from the SwissSidechain database (62). MD simulations were performed using Gromacs, version 5.0.4 (https://www.gromacs.org/). Initially, up to 50,000 steps of steepest-descent energy minimization were performed to relax the system. This was followed by a 1 ns equilibration phase employing both NVT and NPT ensembles. Subsequently, a 500 ns production MD run was carried out for each system at a stable temperature of 300 K, with a time step of 2 fs. For interaction calculations, a 10 Å cutoff was used for short-range interactions, and the particle-mesh Ewald method was applied to manage long-range interactions. MD trajectories were analyzed using the Gromacs tools, along with MDAnalysis and scikit-learn Python libraries (44, 45). Protein–protein contacts were obtained using the mdciao (https://github.com/gph82/mdciao) and GetContacts (https://getcontacts.github.io/) python libraries. Visualization of the results was performed using PyMOL (https://www.pymol.org/) and the Matplotlib Python library (https://matplotlib.org/).

### RNA-Seq and analysis

Total RNA isolation from WT and S7A cells was performed using the SV Total RNA Isolation System (Promega) as per the manufacturer’s provided protocol. Library preparation and sequencing were performed at Bionivid Technology Private Limited using the Illumina NovaSeq 6000 System (Illumina). Adapters and low-quality reads were trimmed using Trimmomatic, v0.39 (63). The quality of the processed reads was evaluated using FastQC, and *de novo* transcriptome assembly was carried out with Trinity v2.15.1 under default parameters. To verify strand specificity, the reads were aligned back to the Trinity assembly using Bowtie 2, and the distribution of strand-specific RNA-Seq data was examined by analyzing the orientation of paired-end fragment reads. CD-HIT-EST (version 4.7) was employed to cluster nucleotide sequences and remove redundant or near-identical transcripts, using a sequence identity cutoff of 100% while maintaining default settings. Differential expression analysis was conducted using edgeR, v3.0.8 (64), which standardized read counts through scaling normalization. Differentially expressed genes (DEGs) were identified using the DEGseq R package, v1.20.0, with the Benjamini–Hochberg method applied for *p* value adjustment. DEGs were filtered using thresholds of |log2(fold change)| >2 and *Q* value <0.05. Functional annotation of DEGs was performed using the Blast2GO software suite, v4.1. Homology searches were carried out with BLASTX and BLASTN against the nonredundant protein database from the National Center for Biotechnology Information, using default parameters. Protein sequence analysis and classification were conducted with InterPro (EMBL-EBI, https://www.ebi.ac.uk/interpro/).

## Data availability

The data are included in the article and also available upon request. This BioProject accession number is PRJNA1272765.

## Supporting information

This article contains supporting information.

## Conflict of interest

The authors declare that they have no conflicts of interest with the contents of this article.
