# Supplementary material for: The Ser7 phosphorylation of RNA polymerase II-CTD is required for the recruitment of E3 ubiquitin ligase Asr1 and subtelomeric gene silencing
Source: J Biol Chem. 2025 Jun 11;301(7):110365. doi: 10.1016/j.jbc.2025.110365 (PMC12270679; doi:10.1016/j.jbc.2025.110365)
Supplement: Supplementary Material [file mmc1.docx]

**Supporting information**

**The Ser7 phosphorylation of RNA polymerase II-CTD is required for the recruitment of  E3 ubiquitin ligase Asr1 and subtelomeric gene silencing**

Nikita Sudarshan^1,2,$^, Mohd. Asalam^1,$^, [Ashutosh Kumar](https://pubmed.ncbi.nlm.nih.gov/?term=Kumar+A&cauthor_id=32997990)^3#^, Neha Singh^1^, Adity Gupta^1,2^, Ishita De^1,2^, Sanjeev Kumar Singh^4‡^, [Kam Y J Zhan](https://pubmed.ncbi.nlm.nih.gov/?term=Zhang+KYJ&cauthor_id=32997990)g^3§^, and Md. Sohail Akhtar^1,2*^

1. §Biochemistry and Structural Biology Division, CSIR-Central Drug Research Institute, Jankipuram Extension, Sitapur Road, Lucknow, 226031, India.
2. Academy of Scientific and Innovative Research (AcSIR), Ghaziabad- 201002, India.
3. Laboratory for Structural Bioinformatics, Center for Biosystems Dynamics Research, RIKEN, 1-7-22 Suehiro, Tsurumi, Yokohama, Kanagawa 230-0045, Japan.
4. Department of Data Sciences, Centre of Bio Medical Research, Lucknow, 226014, India.

^*^Correspondence: [sohail@cdri.res.in](mailto:sohail@cdri.res.in)


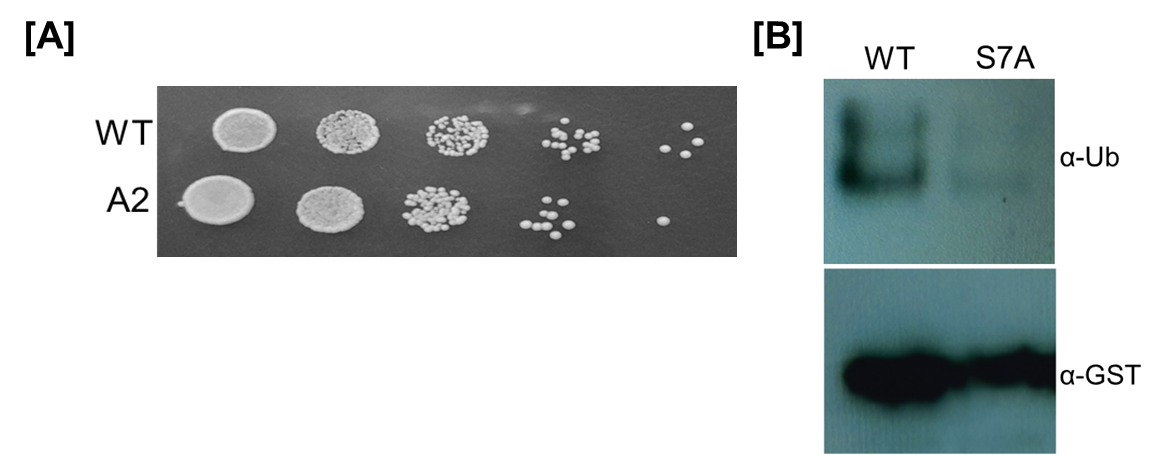


**Figure S1. (A)**  In this experiment, 5 μl aliquots were spotted from the serial dilutions for WT and A2 cells. **(B)** The ubiquitination of consensus (WT) and Ser7 mutant (S7A) GST-CTD by Asr1 and probed against anti-ubiquitin antibody (upper panel). An about equal concentration of both the substrate was loaded for the assay (lower panel).

**
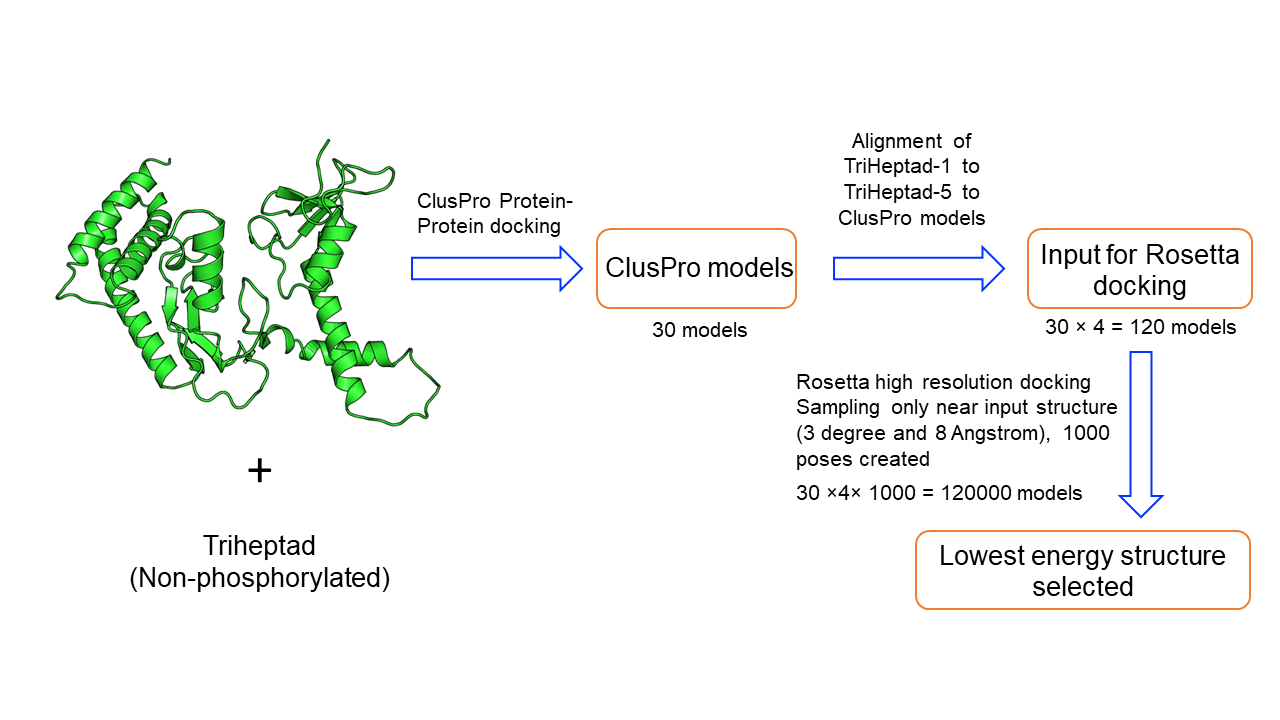
**

**Figure S2.** Multistep protein-protein docking pipeline to predict the interaction of Asr1 with CTD TriHeptads.


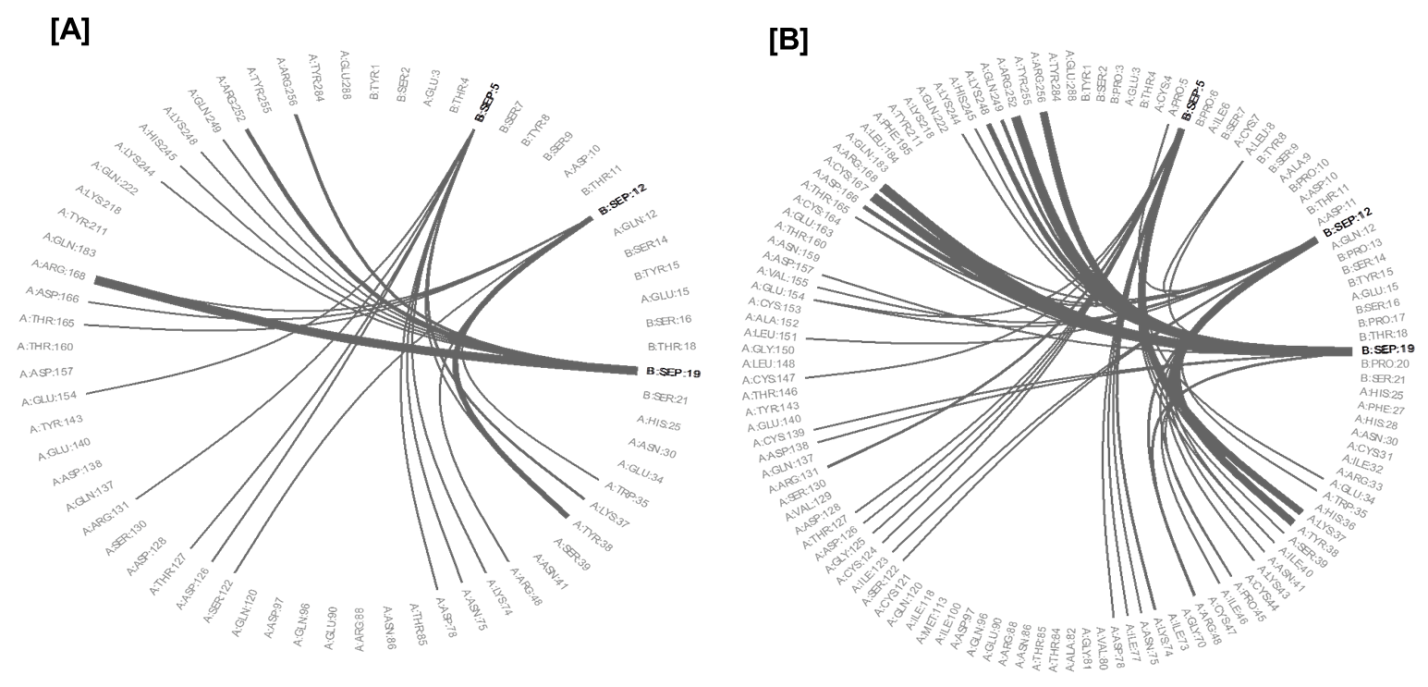


**Figure S3.** Intermolecular hydrogen bonds **(A)** and van der Waals contacts **(B)** calculated between Asr1 and S5-S5-S5 throughout 500 ns MD trajectory. Thickness of line represents higher contact occupancy in 500 ns MD simulation. Contacts were visualized using Flareplot (<https://gpcrviz.github.io/flareplot/>).

**
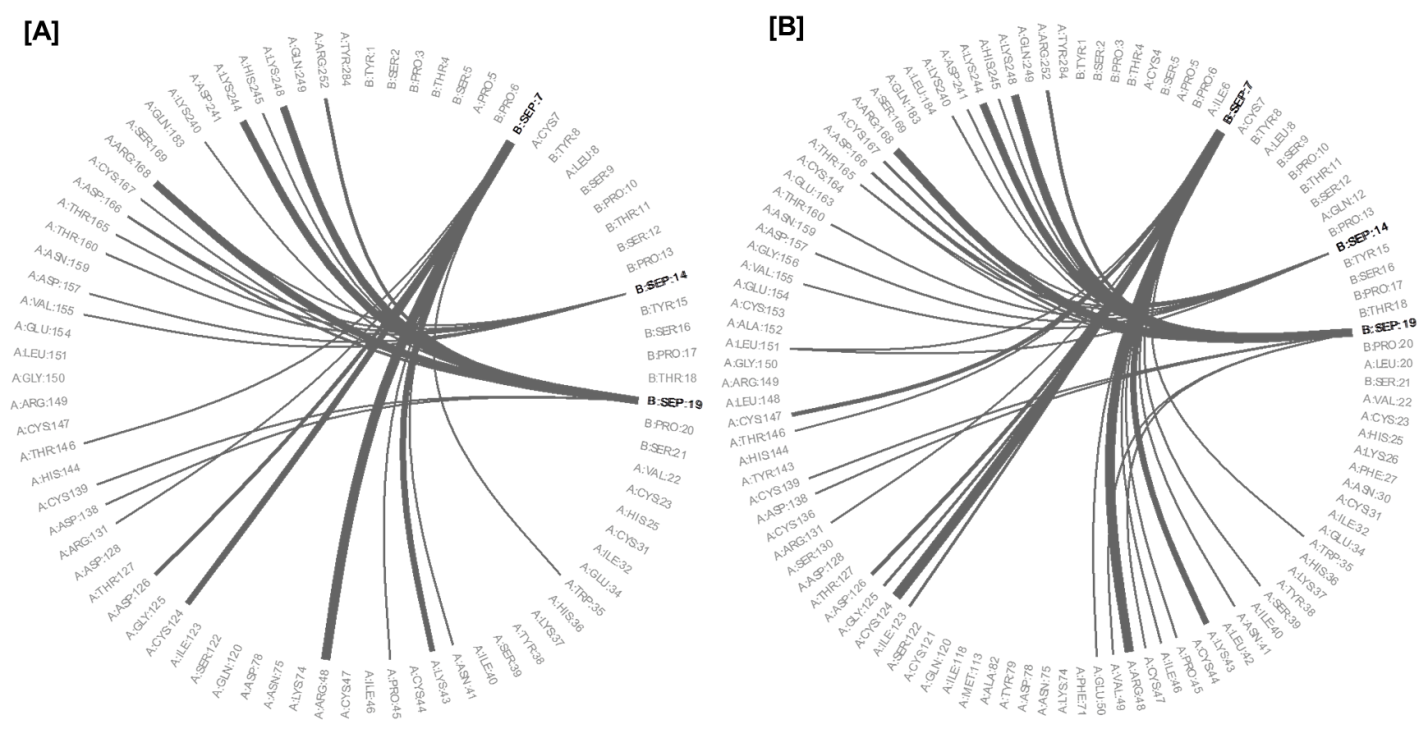
**

**Figure S4.** Intermolecular hydrogen bonds **(A)** and van der Waals contacts **(B)** calculated between Asr1 and S7-S7-S5 throughout 500 ns MD trajectory. Thickness of line represents higher contact occupancy in 500 ns MD simulation. Contacts were visualized using Flareplot (<https://gpcrviz.github.io/flareplot/>).

**
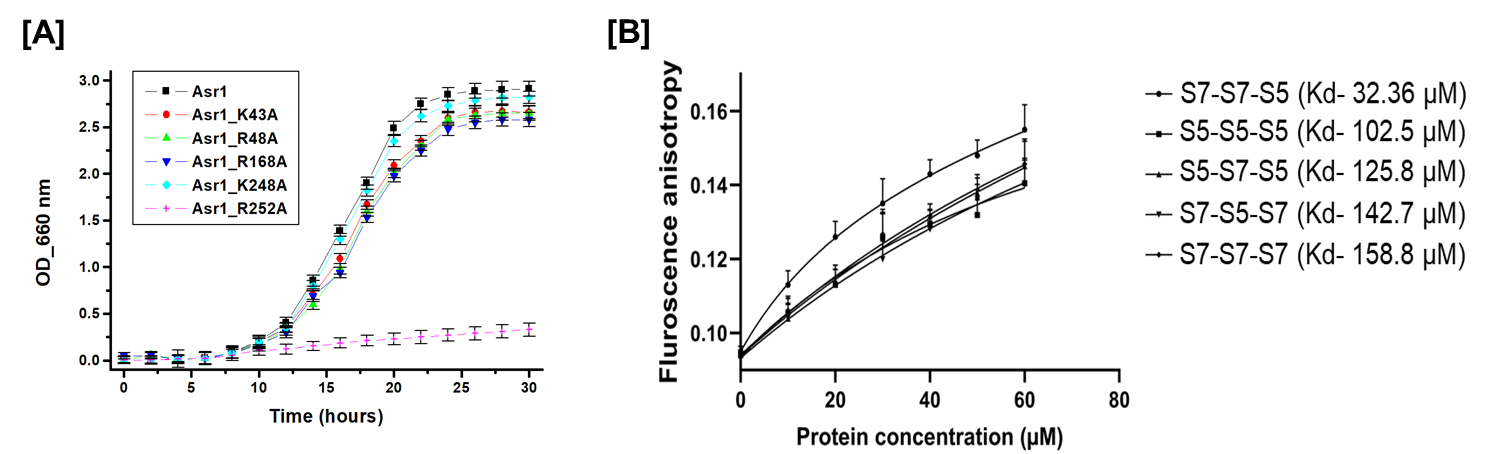
**

**Figure S5. (A)** The growth curve of pJ69-4A strain co-transformed with GAD-CTD and GBD-Asr1 or its various mutants in SC media lacking Leu, Ura and His **(B)** The fluorescence anisotropy measurements, where 2μM of FAM labeled peptides were titrated against increasing concentrations of Asr1 to determine the binding efficiency.

**
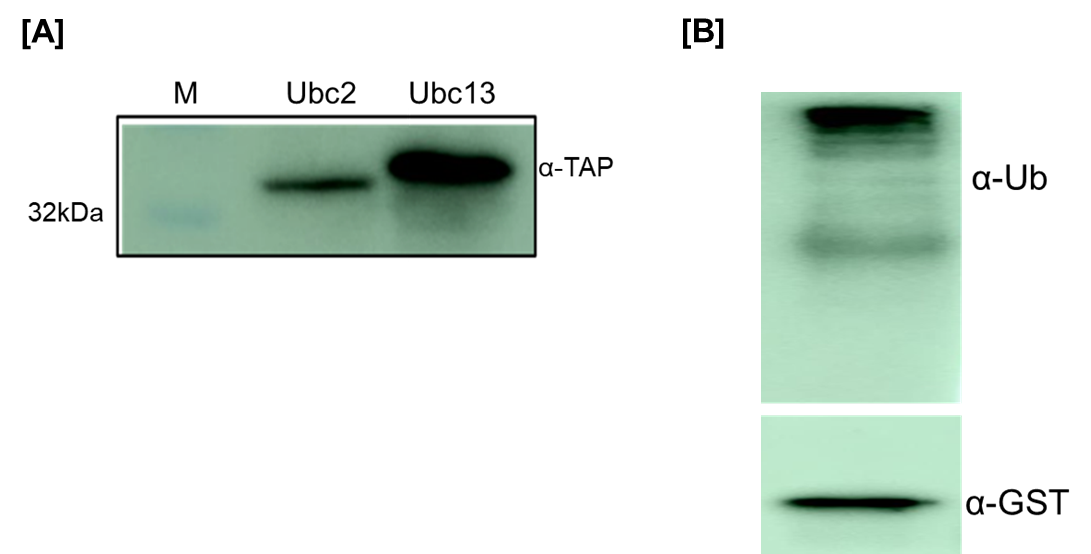
**

**Figure S6.**  **(A)** The detection of purified TAP tagged Ubc2 and Ubc13 by western blotting. **(B)** The *in vitro* ubiquitination assay of GST-CTD by Asr1 in the presence of Ubc2.

**Table-S1**

**The genes induced more than two fold between WT and Ser7 mutant cells.**

| **Gene ID** | **Gene Name** | **log2**  **(Fold Change)** | **p Value** | **Chromosome** | **Median distance from telomere, bp** |
| --- | --- | --- | --- | --- | --- |
| YLR223C | IFH1 | 9.580171797 | 5.1095E-248 | chrXII | 496172 |
| YLR295C | ATP14 | 8.608346872 | 9.62139E-40 | chrXII | 356477 |
| Q0085 | ATP6 | 6.510508826 | 0.028452376 | chrMito | - |
| YCL020W | YCL020W | 6.405224609 | 1.24754E-32 | chrIII | 85101 |
| Q0158 | 21S_RRNA | 4.867419694 | 3.03307E-05 | chrMito | - |
| Q0065 | AI4 | 2.65551454 | 0.026140034 | chrMito | - |
| YOR309C | YOR309C | 2.052697803 | 5.70765E-21 | chrXV | 194594 |
| YGL158W | RCK1 | 1.997444185 | 3.08007E-10 | chrVII | 207031 |
| YLR222C-A | YLR222C-A | 1.870791649 | 3.61802E-08 | chrXII | 495941 |
| YHL012W | YHL012W | 1.575449759 | 2.09462E-05 | chrVIII | 78934 |
| YBR040W | FIG1 | 1.506006893 | 0.002558326 | chrII | 316966 |
| YMR049C | ERB1 | 1.476481075 | 6.26993E-14 | chrXIII | 368092 |
| YKR099W | BAS1 | 1.471844194 | 1.12861E-09 | chrXI | 30966 |
| YNL308C | KRI1 | 1.441213656 | 2.09054E-14 | chrXIV | 54120 |
| YML054C | CYB2 | 1.41081492 | 2.58943E-07 | chrXIII | 165531 |
| YDR464W | SPP41 | 1.410351391 | 6.57029E-13 | chrIV | 143062 |
| YGL215W | CLG1 | 1.40808288 | 1.29424E-08 | chrVII | 87979 |
| YOR153W | PDR5 | 1.40709367 | 3.07417E-10 | chrXV | 471451 |
| YCL037C | SRO9 | 1.362420727 | 2.75744E-06 | chrIII | 57372 |
| YHR071W | PCL5 | 1.36180437 | 1.55496E-10 | chrVIII | 237002 |
| YPR018W | RLF2 | 1.354420118 | 5.6968E-10 | chrXVI | 353590 |
| YDR299W | BFR2 | 1.341912389 | 5.50296E-07 | chrIV | 472307 |
| YPL009C | RQC2 | 1.331092078 | 2.34337E-12 | chrXVI | 412247 |
| YGL170C | SPO74 | 1.315059679 | 0.013065228 | chrVII | 184151 |
| YOR345C | YOR345C | 1.313584485 | 0.000342925 | chrXV | 109129 |
| YDR398W | UTP5 | 1.312791366 | 9.46673E-11 | chrIV | 264463 |
| YNL201C | PSY2 | 1.312371483 | 6.12523E-10 | chrXIV | 260625 |
| YDR464C-A | YDR464C-A | 1.305391641 | 4.63948E-05 | chrIV | 139441 |
| YNR053C | NOG2 | 1.304158217 | 2.17214E-09 | chrXIV | 63213 |
| YMR120C | ADE17 | 1.299038751 | 3.91396E-10 | chrXIII | 416930 |
| YPL012W | RRP12 | 1.284332967 | 6.81563E-10 | chrXVI | 418344 |
| YAL034C-B | YAL034C-B | 1.281301715 | 0.001126759 | chrI | 79487 |
| YBL042C | FUI1 | 1.268136419 | 3.51036E-07 | chrII | 138339 |
| YEL065W | SIT1 | 1.264793821 | 3.60813E-08 | chrV | 27656 |
| YBL024W | NCL1 | 1.253147621 | 1.01287E-09 | chrII | 172533 |
| YAL037C-A | YAL037C-A | 1.249816362 | 0.01215018 | chrI | 73424 |
| YDL062W | YDL062W | 1.240285303 | 0.001402657 | chrIV | 339856 |
| YOR310C | NOP58 | 1.24000994 | 4.5855E-11 | chrXV | 194466 |
| YBR155W | CNS1 | 1.238159884 | 1.24497E-08 | chrII | 263414 |
| YGL120C | PRP43 | 1.235350704 | 4.1441E-10 | chrVII | 281633 |
| YBL054W | TOD6 | 1.2307186 | 9.27979E-07 | chrII | 117588 |
| YML043C | RRN11 | 1.230703854 | 0.00010084 | chrXIII | 190242 |
| YML090W | YML090W | 1.230590255 | 0.012428942 | chrXIII | 90742 |
| YDL063C | SYO1 | 1.220734244 | 2.29345E-07 | chrIV | 338270 |
| YBR263W | SHM1 | 1.218980212 | 2.92838E-10 | chrII | 76920 |
| YMR011W | HXT2 | 1.2115659 | 5.14628E-07 | chrXIII | 288077 |
| YNL091W | NST1 | 1.206487201 | 5.42425E-10 | chrXIV | 331926 |
| YBR142W | MAK5 | 1.204359807 | 3.81771E-09 | chrII | 284868 |
| YGR251W | NOP19 | 1.203558078 | 9.01955E-06 | chrVII | 95301 |
| YCR072C | RSA4 | 1.200154049 | 2.41368E-05 | chrIII | 75816 |
| YLR073C | RFU1 | 1.190769451 | 0.001534094 | chrXII | 281017 |
| YHR153C | SPO16 | 1.188489567 | 0.013993643 | chrVIII | 160558 |
| YER056C | FCY2 | 1.187147611 | 7.37495E-07 | chrV | 266511 |
| YMR076C | PDS5 | 1.187132409 | 3.21886E-06 | chrXIII | 416194 |
| YCR010C | ADY2 | 1.179841185 | 0.011396865 | chrIII | 132272 |
| YKL184W | SPE1 | 1.179736636 | 2.01139E-08 | chrXI | 96756 |
| YLR430W | SEN1 | 1.178311875 | 4.02359E-09 | chrXII | 84743 |
| YOR342C | YOR342C | 1.168465167 | 8.12737E-08 | chrXV | 124626 |
| YPL146C | NOP53 | 1.167491115 | 3.08286E-09 | chrXVI | 276161 |
| YER137W-A | NA | 1.166743123 | 0.001955707 | chrV | 135502 |
| YNCF0001C | RUF21 | 1.15619113 | 0.003947022 | chrVI | 57814 |
| YOR326W | MYO2 | 1.149146178 | 1.18385E-08 | chrXV | 165570 |
| YMR128W | ECM16 | 1.147270556 | 4.78135E-09 | chrXIII | 400735 |
| YPL201C | YIG1 | 1.147039599 | 0.014223788 | chrXVI | 169768 |
| YOL144W | NOP8 | 1.143819275 | 6.71286E-08 | chrXV | 53096 |
| YHR154W | RTT107 | 1.142575756 | 1.81223E-07 | chrVIII | 159677 |
| YKL050C | LPX2 | 1.140402787 | 3.25706E-06 | chrXI | 323965 |
| YNL062C | GCD10 | 1.139805181 | 5.13601E-06 | chrXIV | 275557 |
| YKR105C | VBA5 | 1.126541905 | 0.000400307 | chrXI | 8101 |
| YPL158C | AIM44 | 1.125307197 | 5.80745E-07 | chrXVI | 252032 |
| YDL031W | DBP10 | 1.121416705 | 8.82598E-08 | chrIV | 394215 |
| YPL105C | SYH1 | 1.115843157 | 5.11974E-09 | chrXVI | 352862 |
| YER127W | LCP5 | 1.112116088 | 0.000489707 | chrV | 162394 |
| YDR227W | SIR4 | 1.110601028 | 1.44546E-07 | chrIV | 614362 |
| YKL082C | RRP14 | 1.104927243 | 1.12383E-08 | chrXI | 280919 |
| YGL056C | SDS23 | 1.103661096 | 5.91478E-07 | chrVII | 396033 |
| YDR384C | ATO3 | 1.098616928 | 1.85187E-07 | chrIV | 290730 |
| YNL227C | JJJ1 | 1.092132402 | 4.20611E-08 | chrXIV | 220643 |
| YDR496C | PUF6 | 1.088315992 | 5.00381E-08 | chrIV | 90501 |
| YFR038W | IRC5 | 1.086505654 | 6.73179E-07 | chrVI | 40782 |
| YPL043W | NOP4 | 1.082679669 | 5.11692E-08 | chrXVI | 469938 |
| YOR308C | SNU66 | 1.077100889 | 8.6516E-07 | chrXV | 196668 |
| YNR051C | BRE5 | 1.068532246 | 2.71069E-08 | chrXIV | 67554 |
| YPL044C | YPL044C | 1.062417138 | 2.94171E-06 | chrXVI | 469924 |
| YER137C | YER137C | 1.061689947 | 0.000441166 | chrV | 135502 |
| YDL152W | YDL152W | 1.059236246 | 2.08918E-05 | chrIV | 182821 |
| YLR136C | TIS11 | 1.059060289 | 5.70252E-05 | chrXII | 415800 |
| YKL071W | OSI1 | 1.058809169 | 0.000478423 | chrXI | 305112 |
| YIL091C | UTP25 | 1.058637071 | 0.000195148 | chrIX | 191028 |
| YNL186W | UBP10 | 1.058570183 | 4.16875E-07 | chrXIV | 289497 |
| YHR088W | RPF1 | 1.056966308 | 7.2449E-06 | chrVIII | 281149 |
| YOR206W | NOC2 | 1.05674984 | 7.73549E-08 | chrXV | 363780 |
| YDL066W | IDP1 | 1.055294649 | 4.45907E-08 | chrIV | 334833 |
| YCL054W | SPB1 | 1.055159484 | 1.83023E-07 | chrIII | 31447 |
| YAL035W | FUN12 | 1.053609384 | 2.33273E-08 | chrI | 76425 |
| YDR312W | SSF2 | 1.050488857 | 2.71606E-05 | chrIV | 444353 |
| YOR195W | SLK19 | 1.050107953 | 3.52978E-06 | chrXV | 378426 |
| YML071C | COG8 | 1.048533598 | 4.29319E-07 | chrXIII | 129747 |
| YNL119W | NCS2 | 1.042465602 | 0.000279096 | chrXIV | 383293 |
| YLR226W | BUR2 | 1.041091571 | 2.50735E-05 | chrXII | 488823 |
| YNL002C | RLP7 | 1.04003117 | 1.09043E-07 | chrXIV | 158159 |
| YKR024C | DBP7 | 1.038085995 | 0.000131622 | chrXI | 181673 |
| YDR060W | MAK21 | 1.037703373 | 4.08206E-07 | chrIV | 570648 |
| YDR075W | PPH3 | 1.036240458 | 0.000418538 | chrIV | 597154 |
| YOR337W | TEA1 | 1.03350813 | 0.000395596 | chrXV | 136948 |
| YCL058C | FYV5 | 1.031336693 | 0.001842363 | chrIII | 23522 |
| YHR023W | MYO1 | 1.029462435 | 5.33744E-06 | chrVIII | 151664 |
| YOL091W | SPO21 | 1.029418589 | 0.014036051 | chrXV | 145333 |
| YIL096C | BMT5 | 1.021678294 | 0.000223072 | chrIX | 182115 |
| YGR280C | PXR1 | 1.017241577 | 1.2519E-07 | chrVII | 40030 |
| YHR066W | SSF1 | 1.01720112 | 1.2255E-05 | chrVIII | 229333 |
| YDL227C | HO | 1.015799136 | 0.00111756 | chrIV | 46269 |
| YFL023W | BUD27 | 1.014557026 | 2.29689E-05 | chrVI | 90985 |
| YER165W | PAB1 | 1.012136159 | 1.20809E-07 | chrV | 66502 |
| YCR057C | PWP2 | 1.01040914 | 7.1517E-07 | chrIII | 96163 |
| YNL226W | YNL226W | 1.008032396 | 0.000267182 | chrXIV | 220643 |
| YKL055C | OAR1 | 1.005728048 | 0.015317689 | chrXI | 331496 |
| YOL141W | PPM2 | 1.003697306 | 0.008088507 | chrXV | 56451 |
| YDL148C | NOP14 | 1.003586565 | 2.71087E-07 | chrIV | 188153 |

**Table-S2**

**Strains and plasmids used in this study.**

| **Strain/Plasmid** | **Genotype/Description** | **Source** |
| --- | --- | --- |
| BY4741 | *MAT*a *his3*Δ*1 leu2*Δ*0 met15*Δ*0 ura3*Δ*0* | Our Lab |
| Asr1 TAP | *BY4741, asr1::asr1-TAP KanMX6* | This Study |
| Ubc2 TAP | *BY4741, ubc2::ubc2 –TAP KanMX6* | Horizon discovery |
| Ubc13 TAP | *BY4741, ubc13::ubc13 –TAP KanMX6* | Horizon discovery |
| WT Rpb1 TAP | *JTY1, rpb1::rpb1-TAP KanMX6* | Our Lab |
| S7A Rpb1 TAP | *JTY1, rpb1::rpb1 with 26 repeats YSPTSPA-TAP KanMX6* | Our Lab |
| WT TAP Asr1Δ | *BY4741,* asr1Δ::URA in WT TAP strain | This Study |
| S7A TAP Asr1Δ | *BY4741,* asr1Δ::URA in S7A TAP strain | This Study |
| Asr1 3X FLAG-WT Rpb1 | *WT Rpb1 TAP, asr1::asr1-3X FLAG hphMX6* | This Study |
| Asr1 K43A 3X FLAG-WT Rpb1 | *WT Rpb1 TAP, asr1:: asr1 K43A-3X FLAG hphMX6* | This Study |
| Asr1 R48A 3X FLAG-WT Rpb1 | *WT Rpb1 TAP, asr1::asr1 R48A -3X FLAG hphMX6* | This Study |
| Asr1 R168A 3X FLAG-WT Rpb1 | *WT Rpb1 TAP, asr1::asr1 R168A-3X FLAG hphMX6* | This Study |
| Asr1 K248A 3X FLAG-WT Rpb1 | *WT Rpb1 TAP,asr1::asr1 K248A-3X FLAG hphMX6* | This Study |
| Asr1 R252A 3X FLAG-WT Rpb1 | *WT Rpb1 TAP, asr1::asr1 R252A-3X FLAG hphMX6* | This Study |
| Asr1 3X FLAG-S7A Rpb1 | *S7A Rpb1 TAP, asr1::asr1-3X FLAG hphMX6* | This Study |
| Asr1 K43A 3X FLAG-S7A Rpb1 | *S7A Rpb1 TAP, asr1::asr1 K43A-3X FLAG hphMX6* | This Study |
| Asr1 R48A 3X FLAG-S7A Rpb1 | *S7A Rpb1 TAP, asr1::asr1 R48A-3X FLAG hphMX6* | This Study |
| pMAL-c4X | MBP is expressed under tac promoter | ADDGENE |
| pMAL-c4X-Asr1 | pMAL-c4X:Asr1 | This Study |
| pMAL-c4X-Asr1 K43A | pMAL-c4X:Asr1 (K43A) | This Study |
| pMAL-c4X-Asr1 R48A | pMAL-c4X:Asr1 (R48A) | This Study |
| pMAL-c4X-Asr1 R168A | pMAL-c4X:Asr1 (R168A) | This Study |
| pMAL-c4X-Asr1 K248A | pMAL-c4X:Asr1 (K248A) | This Study |
| pMAL-c4X-Asr1 R252A | pMAL-c4X:Asr1 (R252A) | This Study |
| pGBDU-C1 | DNA binding domain of Gal4p | Our Lab |
| pGBDU-C1-Asr1 | pGBDU-C1:Asr1 | This Study |
| pGBDU-C1-Asr1 K43A | pGBDU-C1:Asr1 (K43A) | This Study |
| pGBDU-C1-Asr1 R48A | pGBDU-C1:Asr1 (R48A) | This Study |
| pGBDU-C1-Asr1 R168A | pGBDU-C1:Asr1 (R168A) | This Study |
| pGBDU-C1-Asr1 K248A | pGBDU-C1:Asr1 (K248A) | This Study |
| pGBDU-C1-Asr1 R252A | pGBDU-C1:Asr1 (R252A) | This Study |
| pGAD-C1 | Transcriptional activation domain of Gal4p | Our Lab |
| pGAD-C1-WT | pGAD-C1:26 repeats of YSPTSPS | Our Lab |
| pGAD-C1-S2A | pGAD-C1:18 repeats of YAPTSPS | Our Lab |
| pGAD-C1-S5A | pGAD-C1:15 repeats of YSPTAPS | Our Lab |
| pGAD-C1-S7A | pGAD-C1:16 repeats of YSPTSPA | Our Lab |

**Table-S3**

**Primes used for cloning of Asr1 and its mutants.**

| **Primer Name** | **Orientation** | **Sequence** |
| --- | --- | --- |
| Asr1 FP-BamH1 | Forward | CGGGATCCATGGAAGAGTGTCCTATT |
| Asr1-RP Sal | Reverse | ACGCGTCGACTTCATCATGACAATAAAT |
| Asr1 K43 A FP | Forward | CAACTTGGCGTGCCCCAT |
| Asr1 K43 A RP | Reverse | ATGGGGCACGCCAAGTTG |
| Asr1 R48A FP | Forward | CATCTGCGCGGTTGAATCCAC |
| Asr1 R48 A RP | Reverse | GTGGATTCAACCGCGCAGATG |
| Asr1 R168A FP | Forward | GCACAGATTGTGCATCAAATGCAT |
| Asr1 R168A RP | Reverse | ATGCATTTGATGCACAATCTGTGC |
| Asr1 K248 AFP | Forward | ACACGTCAACGCGCAAGTATC |
| Asr1 K248 A RP | Reverse | GATACTTGCGCGTTGACGTGT |
| Asr1 R252A FP | Forward | CAAGTATCCGCCAAGTTGTACCG |
| Asr1 R252A RP | Reverse | CGGTACAACTTGGCGGATACTTG |

**Table-S4**

**Primes used for Asr1 gene deletion.**

| **Primer Name** | **Orientation** | **Sequence (5’-3’)** |
| --- | --- | --- |
| Asr1 Del 5’HR FP-BamH1 | Forward | CGGGATCCTTATTAGATCAAACTG |
| Asr1 Del 5’HR RP-BamH1 | Reverse | CTTATATGTAGCTTTCGACATCTTTTTATATGATA |
| 5’HR+URA Del FP | Forward | TATCATATAAAAAGATGTCGAAAGCTACATATAAG |
| 5’HR+URA Del RP | Reverse | TGTTATATTGCATTATTAGTTTTGCTGGCC |
| Asr1 Del 3’HR FP-Spe1 | Forward | GGCCAGCAAAACTAATAATGCAATATAACA |
| Asr1 Del 3’HR RP-Spe1 | Reverse | GGACTAGTAATCTGCCTCTTTGTAG |

\

**Table-S5**

**Primers used for construction of Asr1 Flag tag strain.**

| **Primer Name** | **Orientation** | **Sequence (5’-3’)** |
| --- | --- | --- |
| Asr1 5’HR FP | Forward | CGGGATCCCTATCCAGTCTATTGCTGCCAAGGTTT |
| Asr1 5’HR+Asr1 RP | Reverse | CAAATAGGACACTCTTCCATCTTTTTATATGATATTTGGA |
| Asr1 overlap FP | Forward | TCCAAATATCATATAAAAAGATGGAAGAGTGTCCTATTTG |
| Asr1 overlap RP | Reverse | TCACCGTCATGGTCTTTGTACTCATCATGACAGTAAATTA |
| Asr1 3X FLAG FP | Forward | TAATTTACTGTCATGATGAGTACAAAGACCATGACGGTGA |
| Asr1 3X FLAG RP | Reverse | TTGGCGCGCCCTAATAAGAAATTCGCTTATTTAGAA |
| Asr1 3’HR FP | Forward | CGAGCTCAACAGTATAATACTCTAGTATGAGCCC |
| Asr1 3’HR RP | Reverse | GGACTAGTAATTAAAAATTCCATTGCAGGCAT |

**Table-S6**

**Primers used in ChIP-qPCR study.**

| **Gene** | **Primers** | **Orientation** | **Sequence (5’-3’)** |
| --- | --- | --- | --- |
| irc5 | IRC5 FP | Forward | CGATACTTTACTACATCGAT |
|  | IRC5 RP | Reverse | AGAACGAAGATACAACCACC |
| fyv5 | FYV5 FP | Forward | CGATCTGCTTCAGCATGCTG |
|  | FYV5 RP | Reverse | GCAGCGTCGGTATCATTGTT |
| bas1 | BAS1 FP | Forward | TCACACAATCCCGCTGACAA |
|  | BAS1 RP | Reverse | ATGATGCCGTCGAACTTGGT |
| kri1 | KRI1 FP | Forward | CGAGGATCCCGAATCTGCTG |
|  | KRI1 RP | Reverse | CACGTTGTTGCGAGACGAAA |
